# Healthy Living and Recreation

**Published:** 1922-08

**Authors:** George Wood Clapp


### HEALTHY LIVING AND RECREATION

BY GEORGE WOOD CLAPP, D.D.S.

You have never told me the age of the dentist in your mental movie of success, but it is natural that you should picture him as of about your own age. Put out of mind all that you know about his mental characteristics and run the film slowly and study his physical condition. You will note, first of all, that he is not "old for his years." He does his work without undue fatigue; his manner is pleasant, even in the late afternoon; his mind is keen; and he still feels the stimulus of aspiration. It would never occur to you to make him otherwise in a picture of success. These are characteristics of good health.

Now project your picture twenty years into the future. The dentist is not quite so vigorous as in the earlier years, but his mind is still keen, it still looks forward; and his manner is still pleasant. No success picture can be made with a dentist who is half sick, who is crabbed and whose mind has closed in at the top. He may have passed his physical maximum and be going down the hill, but you feel that he is being forced down, fighting with a smile and yielding only slowly. He may be old and little but he's game. And his eye seems to say that though his body may fail, his mind is immortal and always young.

How does this dentist of the picture correspond with the average of our men in real life?

I have never seen any authoritative figures regarding health and length of life of dentists, but some of the insurance actuaries classify the occupation of dentistry as ten per cent. less favorable to life and health than what they call a "normal occupation." Dentistry does not produce first-class insurance "risks." There is an impression that dentists live, on the average, fifteen years less than physicians who are out of doors more. That means that the dentist who would maintain health and efficiency must use a little more than average care to offset the extra hazard of his occupation by relaxation and play.

Let us come at this from another angle. Level-headed scientists are telling us that the normal length of human life should be about one hundred and twenty years; that about thirty years should be spent in preparation for the life work, about seventy years in its execution and that twenty years, on the average, should remain in which to play golf, grow old and die.

How does this compare with the facts as recorded for all people, including dentists? The average length of life is now about fifty-one years, and it has only recently been lengthened to that. We die when we should be only well started on our life work and when we should have, under ideal conditions, half a century of achievement ahead of us.

The scientists tell us much more than merely the possible length of life. They add that the period of good health should be practically coincident with the life, that we should not be seriously ill and that comfort and vigor should mark our days. This is in striking contrast with the records which show that our period of good health is now, on the average, from the ages of eighteen to forty-two, and that of the remaining nine years we can say, with the Psalmist, "We have no pleasure in them," because of physical illnesses, or "drag-along" half-illnesses.

What do the scientists tell us of the method by which we may lengthen our days and prolong our "pep?" Their prescription is easy to give but difficult to put into effect. They say that all we need to do is to avoid the intake or creation of more poisons than the body can eliminate and to eliminate the poisons unavoidably taken in or created.

They do more than tell us. They show by experimental work with organisms of considerable complexity that life and vigor can be prolonged through several times the usual period. In the case of the fruit fly, they have lengthened life to nine hundred times the usual period. Angle-worms have been kept healthy to the age of ten years. Other experiments support the principle of their statements.

Human lives with an authentic duration closely approaching a century are increasing in number and probably will be common in a generation or two. As I recall the story of those whom I know that are very old, I recognize two things: they have been careful about the intake or creation of poisons, and efficient in their elimination. You can tell that by their complexions and appearance.

How does your physical condition compare with that of the dentist in your mental film? You have been long enough in practice to realize that your dreams of mental affluence through the practice of dentistry were not coming true. To meet your needs and accumulate a competency, you lengthened your working hours and when this remedy no longer sufficed, you appeared to be up against a blind wall.

In spite of the work and worry, no serious harm has been done to your physical organism, and your condition is better than that of thousands of men of your own age. This is due far more to your inheritance than to your intelligence.

Oh, yes, I know you take vacations. I remember the one you took at our house. For the first few days, while you were too tired to do anything else, you rested. About the beginning of the second week you began to say that you ought to be back at the office. Then you thought and talked about all the money you were losing by being away. Soon you began to harp upon the cases waiting for you to take care of them. At the end of two weeks nothing could have kept you away longer from your treadmill.

If a short rest like that made you eat as you did the last Sunday you were with us, it is probably better for you to stay at home and work than to take a vacation. Because of some unpleasant experiences of my own, I had been compelled to study the diet question. You may remember joking about what I ate and saying that you didn't see why a man who ate like that should be permitted to eat at all. So I paid particular attention to your dinner. It showed that you were far less tired out from work than you were poisoned out from food and faulty elimination. And this is probably your condition now.

Your dinner that day consisted of a generous portion of chicken, a large helping of potato, some dressing made mostly of bread, two slices of white bread, a sup of coffee with two spoonfuls of sugar and a sweet dessert. You salted and peppered the potato liberally. And you ate as if seventeen patients, each with a thousand dollars in his hand, were demanding your instant attention.

Your conduct during the afternoon was what might have been expected. From about an hour after dinner until bedtime you were practically dead to the world, put out of action by the poisons you had generated, even though you were strong enough for the effects not to appear as acute indigestion. Some day you may be unable to follow that course without discomfort, which will make you glad to correct it.

I'm not going to pose as a food expert, but I'm going to drop just a hint or two which the experts have given me and experience has confirmed, so that, at home or on vacation, you may build up your health rather than break it down. The man who would put them into effect before his system began to break from wrong eating could do a incredible amount of work for many years.

It isn't quite so important how much you eat as how you combine food elements and how well you chew. (Incidentally, this is important as an argument why patients should maintain their masticating efficiency.) You ate too much that Sunday but, far worse, you made food combinations which prevented proper digestion and insured fermentation. Proteids or carbohydrates, taken singly, digest well. Strong proteids and weak carbohydrates go well together. But a strong proteid (chicken) and a strong carbohydrate (potato and bread), when taken together, delay digestion until fermentation sets in. And fermentation means poison, some of which is unavoidably absorbed. The amount of sugar you took in was practically indigestible. A moderate helping of chicken with a lettuce salad and a baked apple, the meal you joshed about, would have been better. You were too badly poisoned to take any exercise, except dozing on the porch, but I played eighteen holes of golf without anything more than pleasant fatigue.

If a vacation means to you only a feeding time, don't take it! Stay at home, eat less, chew it more and be better off physically. But a vacation ought to mean just the opposite. You are to take recreation for purposes of re-creation. And that means not only a minimum intake of self-generated toxins but the elimination of toxins unavoidably stored up in the days of office work.

Your physiology will tell you that you have three great eliminating organs, lungs, skin and kidneys. They function very incompletely under office conditions. That's probably why the death rate of dentists is above normal. Moderate exercise in the open air, golfing, photographing, fishing, all of which are valuable principally for the walking they require, induce activity which, with a proper diet, can be made to eliminate enough poisons to keep one along in pretty good health. But every once in a while one needs a longer rest and a real "housecleaning" and reoxidation to permit the return of health and vigor. The exercise necessary to this can not be taken by riding in an automobile, even if it is a "fliver."

The length of a vacation should be determined by the time required to burn out the suspended poisons and re-establish vigor, not merely by the return of good feeling, which is only an early sign. It usually takes about as long after one feels well to get well as it did to get to where one feels well.

Of course, you will miss some money while you are away, but a properly-taken vacation may enable you to easily make up in February what you missed in August. Observation covering a good many years seems to show that a dentist should not be in his office more than two thousand hours per year and that dentist who puts that two thousand hours into ten months do more work in their lives than those who string it out over twelve months.

Decide in advance what you can afford to spend and what you can do with it. Don't take the office with you. Dismiss from your mind the anxiety of Mrs. A, B and C for your unrivalled services. Half of their impatience is in your mind and the remainder may be in their talk. Probably they will keep you waiting six months after you return.

In any intelligent scheme of office organization or service selling, sound physical health on the part of the dentist is an essential. That means one or more properly taken vacations a year. One of the most successful dentists I know was out of his office one hundred and thirty-three days last year. That means that he had for himself fifty-two Sundays, twelve legal holidays, Saturday and Wednesday afternoons all summer and forty-three days' vacation. But he made every hour in the office count. On the days he was in the office, only about forty-five minutes per day, on the average, were not directly income-producing and they were necessary for office administration. If I could get his health by buying you that sedan you're so crazy about, I'd gladly do it and think it cheap.

I believe that if dentists could be caught young and given proper physical control, including diet and recreation, and taught efficient office administration, they would with perfect comfort, double the number of income hours now averaged by dentists without such control and organization and earn greater remuneration for each hour. They would render a much higher average quality of service and be among the most valuable educators of their communities.

And that would make dentistry a pretty nearly ideal occupation for the high type of men it ought to attract.

So, if you have really set your face toward the organization of the office to produce desirable results, remember that most of the things you are to reorganize are in yourself and that they will require you to be at your best.

Take those two dandy kiddies and have your vacation. Before you realize it they will be old enough to be thinking of taking their vacations with somebody else.

Don't be gone less than a month. Make it longer if you can, and I promise not to bother you again until I get back, about October.—"*Brother Bill*" in *Dental Digest.*

